# The Extensor Pollicis Brevis: A Review of Its Anatomy and Variations

**Published:** 2013-07-01

**Authors:** Shehab Jabir, Harry Lyall, Fortune C. Iwuagwu

**Affiliations:** ^a^St Andrews Centre for Plastic Surgery and Burns; ^b^Department of Orthopaedic Surgery, Broomfield Hospital, Chelmsford, Essex, United Kingdom

## Abstract

**Introduction:** The standard anatomical description of the extensor pollicis brevis tendon provided in textbooks of anatomy is at odds with that of published anatomical studies. It is crucial to the hand surgeon that he or she has a clear understanding of its anatomy, including its variations. The aim of this study was to provide a comprehensive review of the current literature on the anatomy and variants of the extensor pollicis brevis. It is hoped that this review will be indispensable to the hand surgeon in informing him or her about the anatomy and variants encountered when dealing with the extensor pollicis brevis. **Methods:** Inclusion and exclusion criteria were defined and a literature search was carried out on MEDLINE, PubMed, Embase, and Google Scholar from inception to March 2013 for studies on the topic of extensor pollicis brevis anatomy. The following key words were used: “extensor pollicis brevis,” “anatomy,” “anatomic variations,” “cadaveric study,” “clinical study,” “case report,” and “dissection”. **Results:** The search retrieved a total of 52 studies following removal of duplicates. Forty-five studies were excluded following screening of the title and abstract. Three studies were excluded as they did not meet the eligibility criteria, leaving 4 cadaveric studies for inclusion in the review. **Conclusion:** We recommend the use of ultrasound scanning to determine anatomy of the extensor pollicis brevis before reconstructive procedures involving the extensor pollicis brevis, as well as in traumatic injuries to the extensor pollicis brevis. There appears to be ethnicity-related variations in the anatomy of the extensor pollicis brevis, and further study into these variations may be indicated.

The standard anatomical description of the extensor pollicis brevis (EPB) found in current anatomical textbooks describes its origin as the posterior surface of the radius and the adjacent part of the interosseous membrane, distal to the attachment of the abductor pollicis longus (APL). Its tendon is described as inserting into the posterior surface of the base of the proximal phalanx of the thumb after passing under the extensor retinaculum.^1^ Its primary function is described as extending the metacarpophalangeal joint (MCPJ) of the thumb, with contributions to abduction of the thumb and carpus, as well as stabilizing the MCPJ of the thumb by integrating into the extensor hood.^2^^,^^3^

However, Dawson and Barton,^4^ from their study of the EPB on 16 cadaveric hands, concluded that this standard description does not reflect reality and that EPB tendon anatomy varies considerably from the standard description, even between 2 hands of the same individual. It is of importance to understand the anatomy of the EPB when assessing and considering treatment of acute traumatic and chronic conditions affecting this tendon. The EPB is essential to certain reconstructive hand procedures, such as opponensplasty, extensor pollicis longus repair, thumb stabilization, and MCPJ fusion. Furthermore, recently, there has been an increasing appreciation within the surgical anatomical communities of the impact of anatomical variations on clinical examination, investigation, and, most importantly, operative treatment.^5^ In fact, certain reports have suggested that a substantial proportion of clinical malpractice claims within the surgical specialities is the direct result of the operator's ignorance of anatomical variants within the region of operative intervention.^6^ Therefore, an appreciation of the anatomy and variations of the EPB cannot be overemphasized.

The aim of this study was to provide a comprehensive review of the current literature on the anatomy and variants of the EPB. It is hoped that this review will be indispensable to the hand surgeon in informing him or her about the anatomy and variants encountered when dealing with the EPB.

## METHODS

A literature search was carried out on MEDLINE, PubMed, Embase, and Google Scholar from inception to March 2013 for studies on the topic of EPB anatomy. The following key words were used: “extensor pollicis brevis,” “anatomy,” “anatomic variations,” “cadaveric study,” “clinical study,” “case report,” and “dissection”. The search terms were combined with the Boolean operator “and.” The references of selected studies were also perused for articles that may have been missed via the electronic search.

The title and abstract of all identified studies were examined by one reviewer (S.J.) for suitability. In cases where suitability of a study for inclusion in the review was unclear, the entire article was obtained and assessed for suitability. Eligibility was determined by the criteria listed in Table 1. Any issue pertaining to eligibility of studies was solved via discussion with the senior author (F.I.).

## RESULTS

The search retrieved a total of 52 studies following removal of duplicates. Forty-five studies were excluded following screening of the title and abstract. The entire manuscripts of the remaining 7 articles were reviewed to establish suitability for inclusion. Three studies were excluded as they did not meet the eligibility criteria, leaving 4 studies for inclusion in the review.

All 4 studies included were cadaveric studies focused on assessing the anatomy and associated variations of the EPB tendon. A summary of study features is provided in Table 2. Two studies were carried out in the United Kingdom, 1 in Italy, and the other in India. A total of 268 cadaveric hands were dissected across all 4 studies. Features such as the presence of a clearly identifiable EPB muscle belly, size of the EPB tendon, its insertion into the base of the proximal phalanx and/or extensor hood, its extension onto the distal phalanx, and insertion of tendinous slips into the EPB from other sources such as the APL or base of the first metacarpal were studied. A summary of the anatomical features of the EPB assessed in each study is provided in Table 3.

## DISCUSSION

### The first extensor compartment

**Figure 1:**
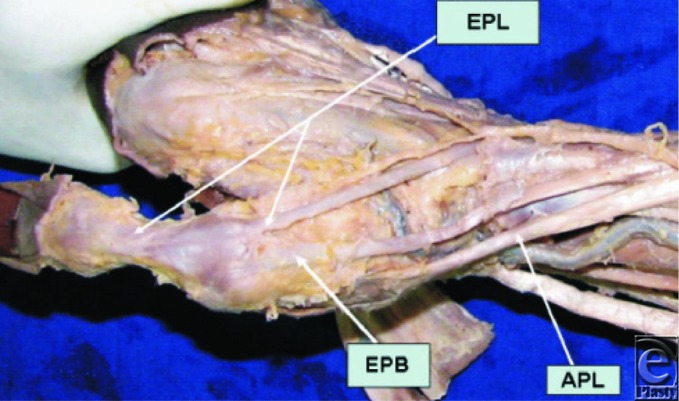
Dorsolateral aspect of the wrist and hand region. APL indicates abductor pollicis longus tendon; EPB, extensor pollicis brevis tendon; EPL, extensor pollicis longus tendon extensor compartment of the wrist. Used with permission from Nayak et al.^7^

Nayak et al^7^ and Brunelli and Brunelli^2^ described the anatomy of the first extensor compartment, while the remaining studies did not assess this aspect. Nayak et al found separate synovial sheaths and compartments for the APL and EPB tendons in 34.6% of cases (54 limbs) within the first extensor compartment. Brunelli and Brunelli, on the contrary, found a similar variation in 9.7% of the cases (5 limbs), with the EPB running in a separate compartment (separated by a fibrous septum from the APL) within the first extensor compartment (see Fig 1).

A number of studies have indicated that the APL and EPB within the first extensor compartment is separated in 30% to 60% of cases by either a complete or partial septum.^5^^,^^8^ This finding has been shown to have implications in the treatment of de Quervain's syndrome. A number of studies have indicated that the presence of multiple osseofibrous compartments may be associated with a greater predisposition to de Quervain's syndrome and may also contribute to the development of de Quervain’s.^9^^-^11 Hence, the ability to recognize the presence of a separate osseofibrous compartment and decompress it has implications in the treatment of de Quervain’s.

### The muscle belly of the EPB

The anatomy of the EPB muscle belly was described in all the 4 studies. Dawson and Barton found that a separate muscle belly for the EPB was absent in 3 hands (23.1%) (ie, the EPB tendon originated from the same muscle belly as the APL tendon) and present in the remaining 13. Brunelli and Brunelli documented the absence of a separate muscle belly in 2 (3.8%) of their 52 hands, while Kulshreshtha et al documented the absence of a separate muscle belly in 3 hands (6.8%) of a total of 44 cadaveric hands. However, Nayak et al documented the presence of a distinct EPB muscle belly in all of the 156 limbs they dissected.

**Figure 2:**
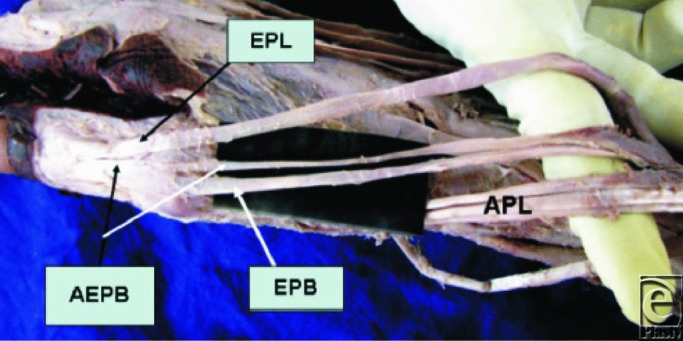
Dorsolateral aspect of the wrist and hand region. APL indicates abductor pollicis longus tendon; EPB, extensor pollicis brevis tendon; EPL, extensor pollicis longus tendon. Note the extensor pollicis brevis has its origin from the abductor pollicis longus muscle. Used with permission from Nayak et al.^7^

The EPB and APL are considered to differentiate from a common muscle mass and are found as completely separate entities only in humans and gorillas.^4^^,^^5^ This theory of a common origin for the EPB and APL is further supported by the fact that, in certain cases, the EPB muscle is fused to a variable extent with the muscle belly of the APL (as documented in Kulshreshtha et al) (see Fig 2). Dawson and Barton, as well as Nayak et al, argue that this is an indication of the phylogenetically young nature of the EPB. However, it can be argued that an absent or abnormally small EPB does not limit movement of the thumb and the use of the EPB in reconstructive procedures has shown that the EPB may in fact be dispensed of without any consequences on the mobility of the thumb.^12^ This, in turn, brings into question the purpose of developing a completely separate muscle that appears to make no clearly identifiable contribution to thumb extensor function. However, a counter argument to this may be that it is precisely because the EPB is at the infancy of evolution that we are as yet unable to precisely understand how it may contribute to thumb function.

### The size of the EPB tendon

Apart from the study by Nayak et al, the remaining 3 studies documented the size of the EPB tendon. However, the method of evaluation of the size of the EPB tendon differed from study to study. Brunelli and Brunelli demonstrated the most objective technique of quantifying size by measuring tendon width. They considered a width of more than 2 mm to be anatomically “normal,” while anything less than this was considered either thin (1-2 mm) or very thin (1 mm). They found that only 10 tendons (19.2%) were of anatomically “normal” diameter with a width of more than 2 mm, the remainder falling into either the category of thin (36.2%, 20 tendons) or very thin (36.2%, 20 tendons). In the study by Dawson and Barton, no measurements of tendon width or thickness were made, rather they used a subjective system of classifying the tendon as thin and of doubtful functional value. This was considered the case in 10 tendons (62.5%). Kulshreshtha et al compared the thickness of the EPB tendon with that of the APL tendon. They found that 16 tendons (36.3%) were less than 33% of the thickness of the APL tendon. They have not elucidated how this comparison was made. Whether the thickness of each EPB was compared with the thickness of the corresponding APL on each cadaveric hand or whether an average value of APL thickness was obtained and comparison of EPB thickness made with this average value is not elucidated. Nor have they explained on what basis this technique was used.

The size of the EPB tendon is an important consideration, as this has implications regarding its suitability for usage in reconstructive procedures on the hand. A small tendon would be of little value in such procedures. However, a small tendon with a correspondingly small muscle belly may be so lacking in strength that its loss in traumatic circumstances may not significantly impact on function. In such cases, repair of the EPB may be not only difficult because of size but also unnecessary because of its lack of functional significance.

### EPB tendon insertion

**Figure 3:**
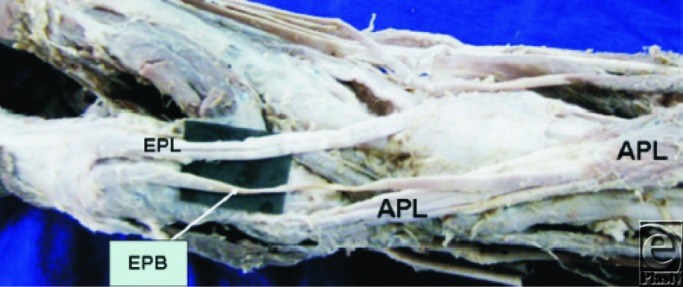
Dorso-lateral aspect of the wrist and hand region. APL indicates abductor pollicis longus tendon; EPL, extensor pollicis longus tendon. The star indicates the extensor hood of the thumb; 3 indicates the tendon of the extensor pollicis brevis; 1 and 2 indicate accessory tendons of the extensor pollicis brevis. Note the insertion of the accessory extensor pollicis brevis tendon 1 to the extensor hood of the thumb. Used with permission from Nayak et al.^7^

As mentioned previously, the EPB tendon is classically described as inserting into the dorsum of the base of the proximal phalanx of the thumb. This, however, appears uncommon in reality from the studies discussed in this review. A detailed study of the insertion site of the EPB was undertaken in all studies apart from Nayak et al. The different insertion patterns and their frequencies are provided in Table 4. Figure 3 provides an example of the insertion of an accessory EPB tendon into the extensor hood of the thumb. It is apparent from Table 4 that the insertion patterns differ quite significantly between each study. There is, however, no clear explanation for this phenomenon.

### Accessory tendons associated with the EPB

**Figure 4:**
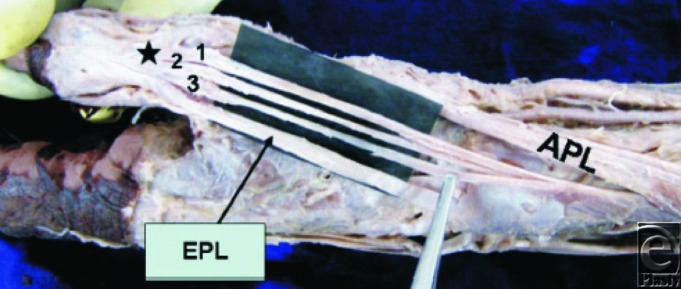
Dorsolateral aspect of the wrist and hand region. AEPB indicates accessory extensor pollicis brevis tendon; APL, abductor pollicis longus tendon; EPB, extensor pollicis brevis tendon; EPL, extensor pollicis longus. Note the insertion of the AEPB to the base of the distal phalanx of the thumb along with the insertion of the EPL. Used with permission from Nayak et al.^7^

Apart from Brunelli and Brunelli, all other studies documented the presence of accessory tendons arising from the EPB (see Figs 3 and 4). These accessory tendons associated with the EPB may arise proximal to, within, or distal to the first dorsal compartment of the wrist. They are commonly described in anatomy textbooks as inserting either into the first metacarpal base or into the extensor pollicis longus. However, Dawson and Barton described 2 (12.5%) cases where accessory tendons from the EPB distal to the first extensor compartment inserted into the APL tendon, while Nayak et al described 11 (7%) limbs where the accessory tendons, again distal to the first extensor compartment, from the EPB inserted into the base of the distal phalanx. Hence, the standard anatomical description requires augmenting to include the possible insertion of accessory tendons from the EPB into the APL as well as the distal phalanx. Furthermore, Kulshreshtha et al confirmed Dawson and Barton's finding by describing the presence of accessory tendons between the EPB and APL in 2 (4.5%) specimens. They also described the presence of accessory tendons from the EPB in 4 (9.1%) other specimens inserting into the base of the first metacarpal as per the standard anatomical descriptions.

An appreciation of the anatomical variants encountered in terms of accessory tendons from the EPB is necessary when mobilizing the EPB for reconstructive hand surgery procedures. Apart from that, it is also essential to properly recognize accessory tendons when carrying out decompression procedures for de Quervain’s, as leaving unrecognized accessory tendons compressed in their own fibrous canal may lead to continued symptoms associated with de Quervain’s.^5^

## CONCLUSIONS

From the discussion earlier, it is clear that the EPB has a number of variants that differ significantly from the standard description and it is vital that the clinician operating in this region of the hand is aware of these potential variations. Dawson and Barton make the observation in their study that the EPB may have significant differences between the 2 sides of the same subject. This observation was confirmed by both Brunelli and Brunelli and Kulshreshtha et al. This brings into question the usefulness of comparing the EPB in the 2 hands of the same individual either clinically or by ultrasound.

An appreciation of the EPB and its variants is essential to gain a complete understanding of thumb extension; however, it is unclear how important the EPB is to thumb extension. Dawson and Barton describe cases where rupture of the extensor pollicis longus lead to an extension lag at both the interphalangeal joint (IPJ) of the thumb and the MCPJ. They explain that this may have been due to absence of the EPB or by failure of the EPB to extend beyond the middle of the first metacarpal. Furthermore, even if the EPB was present and did insert into the MCPJ, its muscle belly and tendon may be so minute that it may lack the necessary strength to fully extend the first proximal phalanx without the assistance of the extensor pollicis longus. Conversely, they also noted that in some patients, rupture of the extensor pollicis longus tendon did not have a significant impact on interphalangeal joint thumb extension, the explanation in this case being a strong enough EPB muscle and tendon with insertions into the distal phalanx, enabling a sufficient degree of interphalangeal joint extension. It is clear from this study that there appears to be a wide variation in the site of insertion of the EPB. Hence, when assessing dorsal injuries to the thumb with suspected extensor involvement, it is important that one is aware of the variations in tendon size, strength, and insertion site, as this may help gauge the extent of their contribution to thumb extensor movements and from this, it may be possible to determine their importance to thumb function on an individual basis.

Apart from enabling one to assess the extent of traumatic injury in situations where the extensors of the thumb may be involved, knowledge of the anatomy and variations of the EPB is also essential in reconstructive hand surgery. A number of procedures have been described, which make the use of the EPB to reinstate hand function. Among them, Kessler^13^ described transfer of the extensor carpi ulnaris to the EPB to enable thumb opposition, while Harrison et al^14^ described the use of the EPB tendon to repair ruptured extensor pollicis longus tendons secondary to rheumatoid arthritis. Matev^15^ has described displacing the EPB tendon into the carpal tunnel to restore thumb opposition. More recently, Fairhurst and Hansen^16^ have described reconstruction of the ulnar collateral ligament of the MCPJ of the thumb by using the EPB tendon. All of these procedures require an understanding of the possible variations of the EPB that may be encountered, as in certain patients, the EPB may be absent or be too small for the planned procedure precluding its use.

Apart from clinical examination, imaging modalities such as ultrasound may also be used to assess the EPB tendon in situations where clinical assessment alone does not provide sufficient information regarding the tendon. Ultrasound is an accurate, rapid, and cost-effective technique to visualize the EPB tendon.^17^ In situations where a suspected anomaly of the EPB is not clearly visualized by ultrasound scanning, magnetic resonance imaging may be considered the next step.^18^

In the study by Nayak et al, all 156 limbs in the study had a distinct EPB muscle belly, whereas in all the remaining studies, the absence of a separate distinct muscle belly for the EPB occurred in at least some of the specimens. A possible explanation for this is the ethnic difference between the specimens in Nayak et al's study and the remaining 3 studies. Nayak et al used cadavers of Indian origin, while the cadavers for the remaining 3 studies were of European origin. This difference is an important indicator of how ethnicity may influence the frequency of anatomical variations.

It is interesting to note that our search for literature revealed that much time and resources had been dedicated to the study of the first extensor compartment and its anatomical variants because of its association with de Quervain's tenosynovitis. There are a number of papers on this topic, both clinical and cadaveric, originating from a number of different countries. It may be useful for future studies looking at the first extensor compartment to extend their study to include the anatomy and variants of the EPB, as there is currently a need for more studies in this area.

This study has shown the wide variation that exists in the EPB tendon and muscle, which are seldom covered adequately in standard anatomical textbooks for the hand surgeon. This study demonstrates that the EPB is one of the most variable muscles in the forearm, requiring further study.

## Figures and Tables

**Table 1:** Inclusion and exclusion criteria

Inclusion criteria
Human subjects
English language
Cadaveric study
Clinical study
Case reports
Exclusion criteria
Foreign language publications
Studies on the first extensor compartment

**Table 2:** Summary of study features included in this review

Author	Country	No. of cadaveric hands dissected
Dawson and Barton^4^	United Kingdom	16
Brunelli and Brunelli^2^	Italy	52
Kulshreshtha et al^3^	United Kingdom	44
Nayak et al^7^	India	156

**Table 3:** Summary of the anatomical features of the EPB assessed in each study*

Author	Presence of EPB and/or muscle belly	Size of EPB tendon	Insertion site of EPB	Extension into distal phalanx	Presence of tendinous interconnections with EPB
Dawson and Barton^4^	Yes	Yes	Yes	Yes	No
Brunelli and Brunelli^2^	Yes	No	Yes	Yes	No
Kulshreshtha et al^3^	Yes	No	Yes	Yes	Yes
Nayak et al^7^	Yes	Yes	Yes	Yes	Yes

*EPB indicates extensor pollicis brevis.

**Table 4:** Comparison of the frequency of different patterns of insertion of the EPB reported in the literature*†

	Wholly to base of prox phalanx, %	Wholly to extensor hood, %	Partly to base of prox phalanx and partly to extensor hood, %	Partly to base of prox phalanx, partly to extensor hood, then further continuation to distal phalanx, %	Only to extensor hood, then further continuation to distal phalanx, %	Extensor pollicis brevis absent, %
Dawson and Barton,^4^ 16 hands	25	18.7	56.2	0	0	6.2‡
Brunelli and Brunelli,^2^ 52 hands	0	69.2	19.2	7.5	0	3.75
Kulshreshtha et al,^3^ 44 hands	25	2.3	25	27.3	20.5	0
Nayak et al,^7^ 156 hands	85.2	No record	No record	No record	No record	0

*Adapted from Kulshreshtha et al.^3^

†EPB indicates extensor pollicis brevis; prox, proximal.

‡Substituted by a slip of the abductor pollicis longus.
